# Trends of People Using Drugs and Opioid Substitute Treatment Recorded in England and Wales General Practice (1994-2012)

**DOI:** 10.1371/journal.pone.0122626

**Published:** 2015-04-29

**Authors:** Hilary R. Davies, Irwin Nazareth, Irene Petersen

**Affiliations:** University College London Department of Primary Care and Population Health, Rowland Hill Street, London, NW3 2PF, United Kingdom; National Taiwan University, TAIWAN

## Abstract

**Background:**

Illicit drug use is a multifaceted public-health problem with potentially serious impacts. The United Kingdom has one of the highest prevalence of illegal drug use in Europe. Reduction of overall illegal drug use in England and Wales has decreased from 11% to 8.2% (2012/13) over the past 10 years. People who use drugs often seek help from their family doctors.

**Aims:**

To investigate General Practitioners (family doctors) first recording of drug use and opioid substitute treatment in primary care settings.

**Design:**

A descriptive study design. Males and females (16-64 years old) were extracted from The Health Improvement Network (THIN) database.

**Setting:**

England and Wales primary care.

**Method:**

The first recording of drug use and opioid substitution treatment in primary care was estimated for the period (1994-2012). Poisson regressions were conducted to estimate incidence risk ratios (IRR).

**Results:**

We identified 33,508 first recordings of drug use and 10,869 individuals with prescriptions for opioid substitute treatment. Overall, males (IRR 2.02, 95% CI:1.97–2.07), people in the age-group; 16-24 (IRR 6.7, 95% CI:6.4–6.9) compared to those over 25 years and the most deprived (IRR 4.2, 95% CI:3.9–4.4) were more likely to have a recording of drug use. Males (IRR 1.2 95% CI:1.2–1.3), in the age-group; 25-34 (IRR 1.8 95% CI:1.7–1.9) and the most deprived (IRR 3.9 95% CI:3.6–4.3) were the groups more likely to have a opioid substitute treatment prescription.

**Conclusion:**

It is evident from this study that there is little recording of drug use and opioid substitute treatment in primary care. Most drug users do not receive treatment in primary care.

## Introduction

Illicit drug use is a public-health problem with serious impacts[1]. In 2012, between 162 and 324 million people had used an illegal drug globally [2]. The United Kingdom (UK) had the second highest number of illegal drug seizures in Europe in 2012 [3]. According to the Crime survey for England and Wales (CSEW), there has been a reduction in reported drug use from 11% in 2001/02 to 8.2% in 2012/13 [4]. Furthermore there has also been a reduction in problem drug users in treatment and the use of heroin and crack cocaine between 2005 and 2011 [5]. These estimates are based on self-reports and hence may be subject to reporting bias. Moreover, they exclude prisoners, homeless people and students living in halls of residence [4,6,7].

The majority of people use drugs for recreational use and a small proportion become problem drug users and require treatment [5]. In England and Wales, opioid substitute treatment is controlled and therefore more effectively monitored [5]. Thus the Welsh drug misuse database is used to monitor problem drug users in treatment in Wales [8] and in England, The National Treatment Agency (NTA) was established as a special health authority in 2001 and became part of Public Health England (PHE) in April 2013 [5]. Prevention and recovery of drug dependency is incorporated in one of the top five health priorities for PHE 2013/2014 [9]. The National Drug Treatment monitoring System monitors over 1,500 community treatment centres which includes inpatient, outpatient and GP practices in England. Most of the National Treatment Agency services are accessed via self-referral and other referral sources including the NHS, the criminal justice system and GPs [5].

Previous research on recording of drug misuse in general practice using the General Practice Research Database (GPRD) from 1998 to 2005 suggested a decline in recording which could be due to a decrease in overall drug misuse, although it was acknowledged that these results may be influenced by other factors [10]. Another study, also used the GPRD, but examined opiate substitution in primary care and the risk of death from 1990 to 2005 [11]. The results highlighted the need for awareness of increased mortality risk when starting opioid substitute treatment and premature withdrawal of substance abuse medication [11].

In this study we sought to assess more recent changes that may have occurred in recording. We therefore aimed to further describe contemporary GP recording of people who use drugs and opioid substitute treatment in England and Wales and compare these to national community surveys (Crime Survey for England and Wales and National Treatment Agency) using a large primary care database, The Health Improvement Network (THIN).

## Methods

### Ethical Approval

The National Health Service South-East Multicentre Research Ethics Committee granted approval for the THIN scheme to obtain and provide anonymous patient data for research in 2003. The Medical Research Scientific Review Committee (set up in 2009 in the UK) approved this study in 2013 (Approval number:13–026). Cegedim Strategic Data Medical Research UK administers SRC application process.

### Study design and data source

In the UK the majority of individuals (98%) are registered with a general practitioner [12]. In total, 570 general practices contribute their data to THIN which incorporates approximately 6% of the UK population [13]. For this study we used data from the general practices located in England and Wales (n = 460). During consultations, clinical information is entered in the form of standardised Read codes, a hierarchical coding system together with drugs prescribed onto the computer using Vision software [14]. THIN is comparable to the general UK population in terms of demographics, mortality and prevalence of chronic and acute diseases, although under 25s, males and less affluent individuals are slightly underrepresented compared to the national statistics [15]. Social deprivation is recorded in THIN as quintile Townsend scores [16]. Townsend scores are a measure of deprivation in the UK [16]. The score is created by measuring four variables; household number, employment, ownership of car and home to create a single measure of deprivation [17]. A higher score indicates the more deprived areas [17]. The region of the general practice was divided into the former strategic health authorities for England and country for Wales [18]. Different data quality markers and markers for practice computer usage have been developed to ensure recording death acceptable mortality recording (AMR) [19] and acceptable computer usage (ACU) [20] and we applied these in our study.

### Study population and outcome measurements

We extracted information from all individuals who were aged between 16–64 years and permanently registered with a general practice in England and Wales which provided data to THIN between January 1994 and December 2012. Patients are registered for different lengths of time and we therefore use Patient years of exposure as our denominator. We have stratified analysis by age and year. If a person has contributed 10 years of data, they will contribute person years to individual age-bands. An example to illustrate this is if an individual was 18 in 2000 and contributed 10 years of data, the individual will contribute to the 16–24 age-band until 2006 and then contribute to the 35–34 year age band. For each patient a GP can enter a Read code and/or a drug code for a prescription in their computer system. We developed Read code and drug code lists for individuals who use drugs and prescribed opioid substitute treatment according to the methods described by Dave and Petersen, 2009 [21]. We used the following illicit drugs and derivatives of these drugs as search terms: Amphetamine, cannabis, cocaine, crack cocaine, crystal meth, heroin, inhalant, ketamine, khat, Lysergic acid diethylamide, magic mushrooms, mandrax, mephedrone, methadrone, methamphetamine, methylone, naphyrone, nitrites, phencyclidine, polydrug, sedative, solvent [22]. There are both Read codes indicating drug use (n = 530) as well as Read codes for opioid substitute treatment (n = 43). We used the following opioid substitute treatments as search terms for prescriptions; methadone, buprenorphine, naltrexone, naloxone and lofexidine [23,24]. Many drug users receive opioid substitute treatment in the community rather than by their GP [5]. However, the GP may be aware that they receive treatment and thus use these latter Read codes on their computer system. If a Read code had an ambiguous meaning (e.g “drug tolerance”), we excluded it from the list. There were 154 drug codes for prescriptions. For each prescription, we examined the dose for each drug and included those which followed the recommended doses by the National Institute for Health and Care Excellence (NICE), Royal College of General Practitioners (RCGP) and British National Formularies (BNF) for detoxification programmes [25,26,23].

For some individuals, it was not entirely obvious if dihydrocodeine was prescribed for an indication other than to treat substance misuse. In those situations we examined time between prescriptions and individuals were only considered to receive that treatment against illicit drug use if it were prescribed at intervals less than 35 days and dose greater than 450mg.

### Individuals recorded as either using drugs and/or receiving opioid substitution treatment

A first recorded case, was the first record for the individual in a particular calendar year. We identified a group of people based on the first recording of: 1) Read code for either people who use drugs and/or 2) Read code for opioid substitute treatment and/or 3) Prescription for opioid substitute treatment between 1994–2012 and selected the first record for all individuals. We examined the association between people who use drugs and opioid substitute treatment and the following covariates: gender, age, social deprivation and time period.

### Statistical analysis

The socio-demographic profiles were summarised. Recording rates and 95% confidence intervals of a new entry of an individual using drugs or receiving opioid substitute treatment were estimated for each calendar year (1994–2012) taking into account the length of time that each individual was registered with the general practice. Poisson regressions were conducted to calculate the adjusted and unadjusted incidence rate ratios (IRR) of GP recording of people who use drugs and/or opioid substitute treatment. We decided a priori on the basis of the existing literature that age, gender and deprivation were confounders [3,4,10]. We observed an effect modification by gender and therefore stratified the results of age, deprivation and time period by gender. We used a forward stepwise approach and performed likelihood ratio tests to select the most appropriate model. There was no evidence that region improved the fit of the model (p = 0.08) and we therefore excluded this variable from our final model. All data was analysed using STATA (version 13) statistical software (Stata Corp LP, College Station, Texas).

## Results

**Fig 1:**
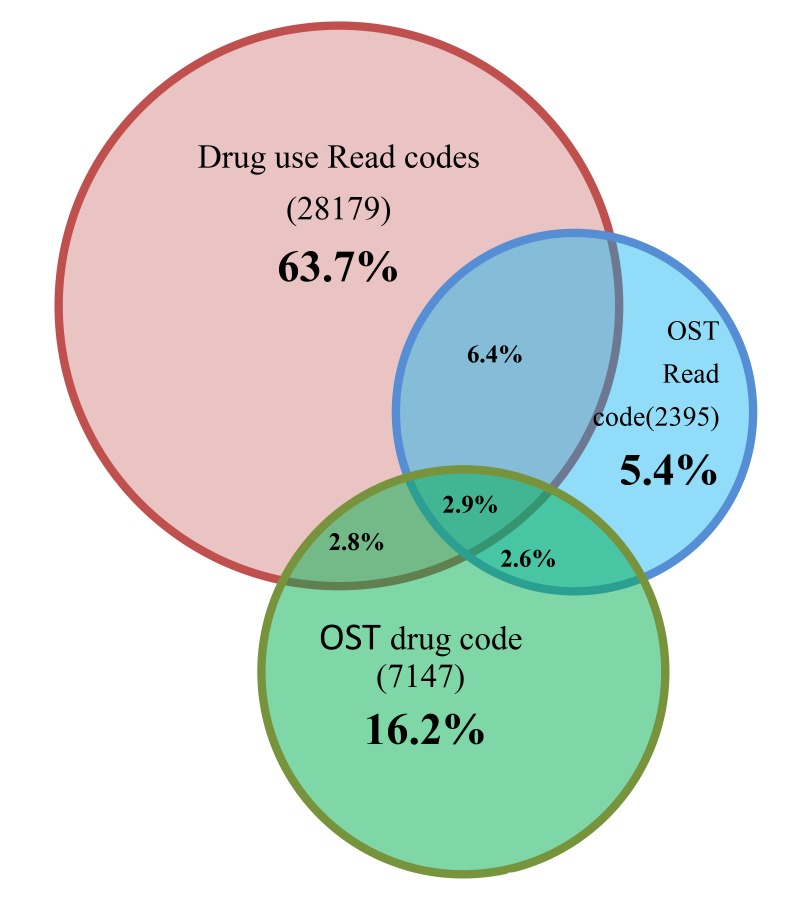
Venn diagram depicting individuals with one, two or three of the Read and/ or Drug codes.

We identified 33,508 individuals with a record of drug use, 10,869 individuals with prescriptions for opioid substitute treatment and 7,655 individuals with Read codes for opioid substitute treatment. GPs used half (51%, n = 267) of the available Read codes for drug use in THIN. A third (35% n = 7) of the 20 most frequently used codes were specific for the illicit drug. Only 18% (n = 27) of the available prescription codes and 72% (n = 31) of the possible Read codes for opioid substitution treatment were used. Patients had different combinations of Read codes and drug codes in their GP computer records (Fig 1), but relatively few individuals had entries of all three. Hence, there were 28,179 (63.7%) individuals recorded as using drugs, but not receiving any opioid substitute treatment in primary care.

Overall there were more males than females with a record of using drugs and/or treatment. Of those with a record for using drugs, most were between the ages of 16–24 years, from the North East of England and of the most deprived group (Table 1). Fewer individuals had Read or drug codes for opioid substitute treatment, but the regional and socio-demographic patterns were similar, although they were slightly older and most of the individuals with a Read code for treatment were from the North West of England (Table 1).

**Table 1:** Demographics, first recording rate (95% CI) for drug use Read codes and Opioid substitution treatment (OST) drug and Read codes by gender, age-band, region and Townsend deprivation score.

	Drug use Read code		Opioid substitution treatment drug code		Opioid substitution treatment Read code	
Demographics	% (n)	Rate (95% CI)	OST drug code	Rate (95% CI)	OST Read code	Rate (95% CI)
		n = 33 508	% (n)	n = 10 869	% (n)	n = 7655
**Gender**						
**Males**	67.5 (22,622)	1.49 (1.40–1.60)	61.8 (6721)	0.45 (0.39–0.51)	68.4(5241)	0.33 (0.28–0.370
**Females**	32.5 (10,886)	0.74 (0.67–0.82)	38.2 (4748)	0.35 (0.29–0.38)	31.6 (2414)	0.50 (0.42–0.56)
**Age-band**						
**16–24**	31.8 (10,650)	2.47 (2.23–2.72)	14.2 (1,542)	0.43 (0.33–0.55)	19.4 (1,482)	0.34 (0.27–0.44)
**25–34**	32.8 (10,994)	1.76 (1.60–1.93)	28.7 (3,122)	0.53 (0.44–0.63)	44.1 (3,377)	0.50 (0.43–0.59)
**35–44**	20.0 (6,704)	0.88 (0.77–0.99)	24.1 (2,624)	0.37 (0.30–0.46)	25.7 (1,970)	0.24 (0.20–0.30)
**45–64**	15.4 (5,160)	0.41 (0.33–0.50)	32.9 (3,581)	0.33 (0.27–0.39)	10.8 (826)	0.06 (0.04–0.08)
**Region**						
**London**	12.1 (4,051)	0.06 (0.05–0.07)	7.25 (788)	0.24 (0.16–0.34)	7.92 (606)	0.16 (0.11–0.24)
**East Midlands**	4.52 (1,514)	0.06 (0.04–0.07)	5.59 (608)	0.46 (0.30–0.69)	5.66 (433)	0.27 (0.17–0.43)
**East of England**	8.26 (2,768)	0.12 (0.11–0.13)	6.90 (750)	0.34 (0.22–0.49)	5.92 (453)	0.16 (0.10–0.26)
**West Midlands**	9.11 (3,051)	0.06 (0.05–0.06)	11.4 (1,243)	0.37 (0.28–0.50)	11.1 (851)	0.22 (0.16–0.31)
**North East**	3.75 (1,258)	0.15 (0.09–0.17)	5.14 (559)	0.64 (0.41–0.96)	4.10 (314)	0.29 (0.18–0.48)
**North West**	16.5 (5,513)	0.10 (0.09–0.11)	15.8 (1,721)	0.54 (0.42–0.68)	21.8 (1,669)	0.46 (0.37–0.57)
**South Central**	11.9 (3,990)	0.12 (0.10–0.15)	3.84 (417)	0.33 (0.25–0.44)	3.14 (240)	0.20 (0.15–0.27)
**South East Coast**	11.5 (3,865)	0.13 (0.12–0.15)	13.4 (1,459)	0.34 (0.24–0.48)	13.9 (1,063)	0.16 (0.11–0.26)
**South West**	9.29 (3,112)	0.05 (0.05–0.06)	10.3 (1,127)	0.46 (0.34–0.61)	7.04 (539)	0.24 (0.17–0.34)
**Yorkshire&Humberside**	3.61 (1,211)	0.05 (0.04–0.07)	12.5 (1,362)	0.35 (0.21–0.56)	10.2 (782)	0.18 (0.10–0.32)
**Wales**	9.48 (3,175)	0.09 (0.08–0.10)	7.68 (835)	0.38 (0.26–0.54)	9.21 (705)	0.27 (0.19–0.40)
**Townsend deprivation**						
**1(least deprived)**	12.0 (3,825)	0.45 (0.38–0.53)	14.3 (1,475)	0.19 (0.15–0.25)	7.21 (526)	0.06 (0.04–0.09)
**2**	13.1 (4,200)	0.65 (0.56–0.76)	15.0 (1,552)	0.26 (0.20–0.34)	9.62 (696)	0.17 (0.11–0.25)
**3**	19.8 (6,321)	1.06 (0.93–1.20)	20.2 (2,092)	0.37 (0.29–0.47)	17.5 (1,275)	0.38 (0.27–0.52)
**4**	26.8 (8,571)	1.68 (1.50–1.88)	26.0 (2,691)	0.58 (0.47–0.71)	29.8 (2,166)	0.77 (0.59–1.02)
**5(most deprived)**	28.4 (9,076)	2.89 (2.59–3.21)	24.4 (2,526)	0.89 (0.72–1.09)	35.8 (2,604)	1.55 (1.24–1.96)

Rate = First recording rate/1000 person years at risk, OST = Opioid Substitution Treatment

### Read codes for individuals who use drugs

Men were twice as likely to have a Read code in their electronic health records for drug use when compared to women (IRR 2.0, 95% CI: 1.9–2.1). For men, compared to the oldest age group, the youngest age group was almost seven times more likely to have a recording for drug use (IRR 6.7, 95% CI: 6.4–6.9) (Table 2).

**Table 2:** Unadjusted and adjusted Incidence Rate Ratios (95% CI) for drug use Read codes by age-band, deprivation, and time period (1994–2012).

Demographics	Unadjusted IRR (95% CI)		*Adjusted IRR (95% CI)	
	Males	Females	Males	Females
**Age-band**				
**16–24**	6.87 (6.58–7.17)	3.97 (3.77–4.19)	6.68 (6.39–6.99)	3.35 (3.17–3.55)
**25–34**	6.60 (6.32–6.89)	3.47 (3.28–3.66)	4.98 (4.76–5.21)	2.09 (1.98–2.22)
**35–44**	3.21 (3.06–3.36)	2.09 (1.97–2.12)	2.29 (2.18–2.41)	1.36 (1.28–1.44)
**45–64**	1	1	1	1
**Townsend Score**				
**1 (least deprived)**	1	1	1	1
**2**	1.45 (1.37–1.53)	1.36 (1.26–1.47)	1.43 (1.36–1.51)	1.32 (1.22–1.43)
**3**	2.20 (2.10–2.31)	2.07 (1.93–2.22)	2.05 (1.95–2.16)	1.88 (1.75–2.02)
**4**	3.37 (3.22–3.54)	3.10 (2.89–3.31)	2.76 (2.63–2.89)	2.48 (2.32–2.66)
**5(most deprived)**	5.61 (5.36–5.88)	5.08 (4.76–5.42)	4.17 (3.98–4.37)	3.69 (3.46–3.95)
**Time periods**				
**1994–2000**	2.30 (2.21–2.39)	2.32 (2.20–2.45)	2.29 (2.21–2.39)	2.28 (2.16–2.41)
**2001–2006**	1.75 (1.70–1.80)	1.69 (1.63–1.77)	1.74 (1.69–1.79)	1.71 (1.64–1.78)
**2006–2012**	1	1	1	1

*Adjusted for age deprivation, and time periods

There was also a trend with regards to social deprivation, men from the most deprived areas were four times more likely to have a drug use Read code recorded compared to those from least deprived area (IRR 4.2, 95% CI:3.9–4.4) (Table 2). Recording of drug use was two times more likely to occur during 1994–2000 compared to 2006–2012 (IRR 2.3, 95% CI: 2.2–2.4). For women, the pattern was similar as for the men, though the contrasts were less stark (Table 2).

### Opioid substitute treatment drug codes

**Fig 2:**
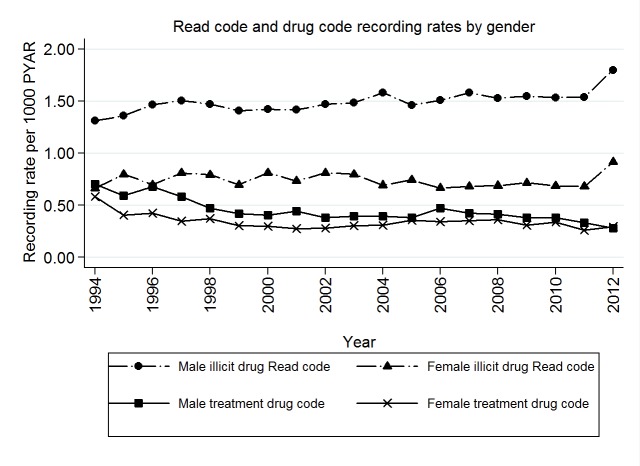
Combined first recording rates of Read (drug use) and Drug (opioid substitution treatment) codes by age-group (in years) per 1000 person years at risk (1994–2012).

There were more individuals with a record for drug use than those actually receiving opioid substitute treatment. (Fig 2).

More men than women received opioid substitute treatment. (IRR 1.2, 95% CI: 1.2–1.3) and also the most deprived group were more likely to have an opioid substitute treatment entry in their notes (Table 3).

**Table 3:** Unadjusted and adjusted Incidence Rate Ratios (95% CI) for opioid substitution treatment drug codes by age-band, deprivation and time period (1994–2012).

Demographics	Unadjusted IRR(95% CI)		*Adjusted IRR(95% CI)	
	Males	Females	Males	Females
**Age-band**				
**16–24**	1.38 (1.27–1.49)	0.89 (0.81–0.97)	1.22 (1.16–1.38)	0.78 (0.71–0.86)
**25–34**	2.72 (2.55–2.91)	1.39 (1.29–1.50)	1.84 (1.71–1.97)	0.86 (0.79–0.93)
**35–44**	1.82 (1.70–1.95)	1.16 (1.08–1.25)	1.58 (1.47–1.71)	0.79 (0.73–0.86)
**45–64**	1	1	1	1
**Townsend Score**				
**1 (least deprived)**	1	1	1	1
**2**	1.40 (1.26–1.54)	1.34 (1.21–1.48)	1.37 (1.23–1.52)	1.31 (1.18–1.46)
**3**	2.07 (1.89–2.27)	1.66 (1.51–1.83)	1.98 (1.80–2.18)	1.59 (1.44–1.76)
**4**	3.10 (2.84–3.38)	2.29 (2.08–2.51)	2.75 (2.51–3.01)	2.08 (1.89–2.29)
**5(most deprived)**	4.86 (4.45–5.30)	3.01 (2.74–3.32)	3.92 (3.58–4.30)	2.55 (2.30–2.82)
**Time periods**				
**1994–2000**	1.84 (1.71–1.97)	2.14 (1.97–2.32)	1.82 (1.69–1.97)	2.01 (1.84–2.19)
**2001–2006**	1.49 (1.41–1.58)	1.62 (1.52–1.73)	1.48 (1.39–1.57)	1.59 (1.49–1.71)
**2006–2011**	1	1	1	1

*Adjusted for age deprivation and time period

**Fig 3:**
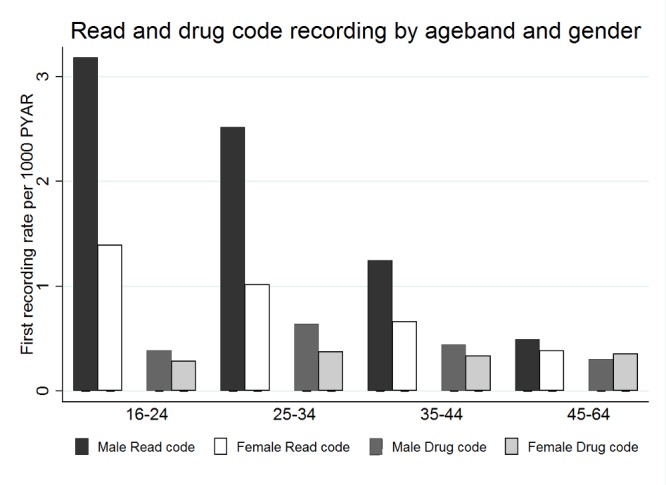
Combined first recording rates of Read (drug use) and Drug (opioid substitution treatment) codes by gender and age-group (in years) per 1000 person years at risk (1994–2012).

Men were slightly older (25–34 years) when they had their first entries for opioid substitute treatment compared to when they had their first record of drug use entered in their electronic health records (Table 3 and Fig 3). Younger women compared to the 45–64 year age group were less likely to have a code for opioid substitute treatment (Table 3).

There has been a decline in people receiving opioid substitute treatment in primary care. Hence, both men (IRR 1.8, 95% CI: 1.7–1.9) and women (IRR 2.0, 95% CI: 1.8–2.2) were significantly more likely to receive a prescription between 1994–2000 compared to the period between 2006–2012.

Similar patterns were found for Read codes for opioid substitute treatment to those of the drug codes (Higher IRR for males, 25–34 age group, most deprived and between 1994–2000) (Table 4).

**Table 4:** Unadjusted and adjusted Incidence Rate Ratios (95% CI) for opioid substitution treatment Read codes by age-band, deprivation and time period (1994–2012).

Demographics	Unadjusted IRR (95% CI)		Adjusted IRR (95% CI)	
	Males	Females	Males	Females
**Age-band**				
**16–24**	4.46 (4.01–4.96)	5.94 (5.14–6.86)	4.64 (4.16–5.17)	4.85 (4.19–5.63)
**25–34**	10.3(9.40–11.31)	9.24(8.09–10.55)	7.87 (7.14–8.67)	5.09 (4.43–5.84)
**35–44**	5.39 (4.89–5.95)	4.05 (3.50–4.68)	3.79 (3.42–4.20)	2.49 (2.15–2.90)
**45–64**	1	1	1	1
**Townsend Score**				
**1 (least deprived)**	1	1	1	1
**2**	1.74 (1.52–1.99)	1.64 (1.34–2.01)	1.69 (1.48–1.94)	1.57 (1.28–1.92)
**3**	3.03 (2.67–3.42)	3.42 (2.86–4.08)	2.72 (2.40–3.08)	2.97 (2.48–3.55)
**4**	5.89 (5.25–6.61)	6.11 (5.16–7.24)	4.66 (4.15–5.24)	4.59 (3.88–5.44)
**5(most deprived)**	11.2(10.0–12.6)	10.9(9.19–12.8)	8.21 (7.33–9.20)	7.51 (6.35–8.88)
**Time**				
**1994–2000**	2.11 (1.94–2.30)	2.33 (2.05–2.65)	2.09 (1.91–2.28)	2.09 (1.91–2.28)
**2001–2006**	1.51 (1.43–1.61)	1.81 (1.66–1.97)	1.49 (1.39–1.58)	1.49 (1.39–1.58)
**2006–2011**	1	1	1	1

*Adjusted for age deprivation and time period

## Discussion

### Summary of main findings

The findings of this study illustrate that there are a number of individuals who are recognised and have a record of drug use. However, if patients required treatment for their drug use, a large proportion of these patients did not receive treatment in primary care. Overall, there were more males than females with records of drug use and of those with a record, most were between the ages of 16–24 years, residing in the North East of England and of the most deprived group. First recording rates were significantly greater for men between the age-groups of 16–24 years (drug use) and 25–34 years (opioid substitute treatment) with high social deprivation and between the period 1994–2000. In order to improve provision of care, we needed to have an understanding of the ongoing current recognition and recording in primary care and how this has changed over time.

### Strengths and Limitations

**Fig 4:**
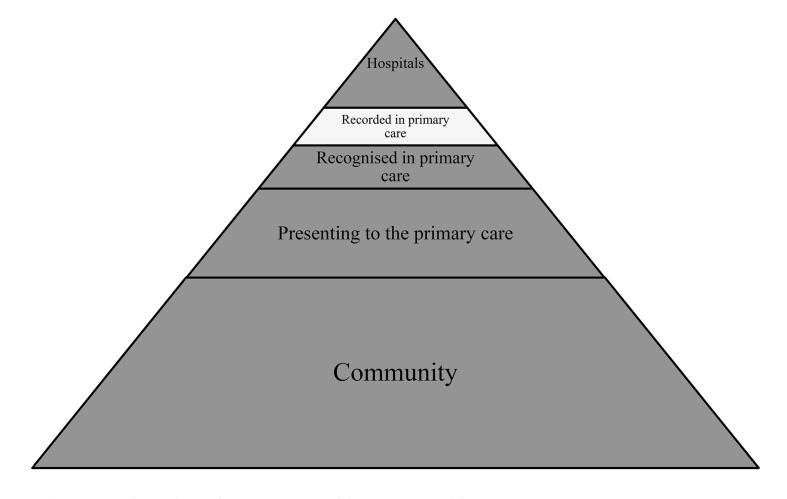
Adaptation of Access to Health care pyramid.

The main strength of this study is that THIN provides a large amount of data from real life primary care. This study, however, merely examines data that has been captured in primary care and is not attempting to estimate a community incidence of the problem. There are some limitations to our study. Firstly this study only covers individuals who have been in contact with their GP in regards to their drug use. Many people use drugs for recreational use and only a small percentage become problem drug users [5]. Drug use is stigmatised and discriminated against in society [27]. This stigma may continue when drug users seek help for either stopping their addiction or for obtaining replacement therapy [27]. Patients, therefore, may decide not to disclose this information to a health professional. Disclosure may however occur when the drug users’ behaviour causes harm and functional impairment [27]. GPs are also aware that the patient-doctor relationship could be compromised if the patient is coded as a drug user [28]. The majority of opioid substitute treatment for drug use occurs in the community drug clinics, only a small proportion reach primary care and even a smaller proportion are recorded electronically [5,29] (See Fig 4). Self-referral (40%) to community drug clinics is more common than GP referral (6%) [5]. Due to patient confidentiality, the patients’ details from self-referrals are not always shared with the GP. The GPs are therefore not always aware when an individual receives treatment and the treatment would not be recorded in their patient records [5].

Secondly, the highest prevalence of drug users are in the following three groups; prisoners, homeless and students. The prisoners and homeless may have difficulty registering with a GP and students may be registered with a different GP during the university term. Data from a 2002 survey found that over 50% of prisoners were not registered with a GP before entering prison [30], 68%, (n = 1276) of English prisoners reported using an illicit drug the year before taken into custody [31]. Drug use can often lead to homelessness and about three quarters of injecting drug users have been homeless at a point in time [7]. With regards to students, approximately 19.8% of 16–24 years old were found to have used an illicit drug (2011/2012) (4). Younger, less affluent males are slightly underrepresented in THIN compared to the national statistics [15].

Thirdly, our results reflect that recording rates are higher for more socially deprived groups. This may be because there are more people using drugs amongst the socially deprived groups, but it could also be masking the fact that drug users with less social deprivation could be seeking help elsewhere (e.g. private health care) or denying the problem and avoiding the stigma associated with drug use [27].

Finally the majority of Read codes used are generic and not specific for a particular drug. This could be because poly-drug use is common amongst drug users [32]. Symptoms are more obvious with opiate drug users whilst symptoms of other drugs are not as obvious and could be missed.

### Comparison with existing literature

#### Gender

With regards to gender, our findings for both drug use and opioid substitute treatment are similar to both the Crime Survey for England and Wales and the National Treatment Agency [4,5]. Since 1996 there have been 50–60% more male illicit drug users [4]. In 2013, 73% of people in treatment were male [5].

#### Age-group

Our results regarding age group showed similar patterns to the Crime Survey for England and Wales, however, the proportion is lower [4]. The younger adults (16–24) were more likely to be recorded using drugs compared to the older age groups.

In our findings the opioid substitute treatment group is higher in 16–24 year olds until 1999 and then there was an increase in 25–34 years age group. The National Treatment Agency has reported that opiate use has decreased significantly in the 16–24 year age group and the percentage of heroin and methadone users has remained unchanged for the last few years [5]. Therefore, those seeking opioid substitute treatment are moving into the older age-groups [33].

#### Social deprivation

Our study illustrates that the higher recording rates of drug use are associated with more social deprivation. The Crime Survey for England and Wales have found an inverse association between frequent drug use and income [4]. The Crime Survey for England and Wales has also reported that frequent drug use is higher in urban areas [4]. Likewise, the opioid substitute treatment is also recorded more in the more socially deprived groups. The National Treatment Agency has reported that those in treatment are more socially deprived.

#### Implications for research and/or practice

Our findings show that recording of both drug use and opioid substitution treatment occurs in primary care, but these figures are lower when compared to those of the Crime survey for England and Wales and the National Treatment Agency. GPs are less likely to record if an individual is requiring treatment but either not presenting to the GP and or going elsewhere to receive treatment. There were approximately 197 110 people in treatment in 2011/12 and 81% (n = 159 659) were being treated from heroine and /or crack use [33]. This suggests that drug use is identified in primary care, but most drug users who need treatment do not receive it in primary care. Currently people are in treatment for General practitioners may be reticent to manage drug dependency in primary care but still play an integral role in the management of patients’ physical health and people who use drugs often have poor physical health [34]. Presently the General Medical Service contract does not have specific Quality and Outcome Framework (QOF) indicators for substance misuse. QOF indicators could be revised to provide incentives to GPs to identify and treat this vulnerable group of people. The RCGP have developed a certified course in the management of drug misuse which can go some way toward helping GPs to handle these problems [35]. The scope to develop clear care pathways for those identified in general practice and then treated either in the practice or referred to an appropriate community drug clinic needs careful consideration. It is possible that not all GPs would wish to engage with people who use drugs leading to a disparity in the provision of care [36]. Nevertheless, appropriate identification of people who use drugs by GPs and appropriate management or referral can go some way in ensuring clarity in the management of this group of people.

We found low level of recording of drug use in general practice notes. It is unclear as why, when and how GPs record sensitive information of this type on the practice computers and whether they use specific Read codes for this purpose. A fuller understanding of this phenomenon would be better investigated through a qualitative study in which a more detailed understanding of the methods, classification and circumstances for recording can be explored together with how to develop a best-practice method of recording drug use on general practice records [37]

## Conclusion

It is evident from this study that there is little recording of drug use and opioid substitute treatment in primary care. Most drug users do not receive treatment in primary care.
